# Mortality, readmission, and reoperation after hip fracture in nonagenarians

**DOI:** 10.1186/s12891-017-1493-5

**Published:** 2017-04-04

**Authors:** Jeff Chien-Fu Lin, Wen-Miin Liang

**Affiliations:** 1 Department of Statistics, National Taipei University, Taipei, Taiwan; 2 Department of Orthopedic Surgery, Wan Fang Hospital, Taipei Medical University, Taipei, Taiwan; 3 Department of Public Health, China Medical University, Taichung, Taiwan

**Keywords:** Hip fracture surgery, Mortality, Complication, Nonagenarians, Readmission, Reoperation

## Abstract

**Background:**

Osteoporotic hip fractures are associated with high mortality and morbidity in people of advanced age; however, few studies have investigated the complication rates in nonagenarians. In this study, we applied a competing risk analysis to estimate the mortality, readmission, and reoperation rates after surgery for hip fracture among nonagenarians.

**Methods:**

A total of 11,184 nonagenarians (aged ≥ 90) who received surgery for hip fracture during the period 1 January 1997 and 31 December 2010 were selected from Taiwan’s National Health Insurance (NHI) database. Nonagenarians were followed up until the end of 2012, death, or the date they left the NHI program. Cumulative mortality was estimated using the Kaplan-Meier analysis and risk factors for mortality were investigated using a Cox proportional hazards model. Competing risk analysis was used to estimate cumulative incidence rates and to assess the risk factors for reoperation and readmission.

**Results:**

The mortality rates were 29.5% at 1 year, 45.0% at 2 years and 78.1% at 5 years. The cumulative incidence rates of reoperation were 7.3% at 1 year, 9.2% at 2 years and 11.6% at 5 years whereas those of readmission were 18.9% at 1 month and 24.1% at 3 months. Significant risk factors for death included age, male gender, trochanteric fracture, and higher Charlson comorbidity index (CCI) whereas those for reoperation were age, cervical fracture and higher CCI. Furthermore, age, male gender, and higher CCI were risk factors for readmission.

**Conclusions:**

The overall 2-years mortality rate among nonagenarians in Taiwan was around 45%, the 2-years reoperation rate was around 9% and the 90-days medical complication rate was around 24%. High complication rates are associated with increased risk for death. Postoperative care to prevent medical complications is likely the most effective strategy to reduce mortality rates among nonagenarians with hip fracture.

## Background

Osteoporotic hip fractures are associated with high rates of mortality and morbidity in patients of advanced age, with 1-year mortality rates approaching 30% in that patient population [1–8]. Although nonagenarians, namely individuals aged 90 years and older, represent a small subgroup, osteoporotic injuries to these individuals pose a great challenge to both surgeons and physicians. One-year mortality rates after hip fracture are as high as 24–50% in nonagenarians [9–26]. In addition, 1-year readmission rates are 5–30% and 2-years reoperation rates may be as high as 20% after surgery for hip fractures in elderly adults [27–35]. Unplanned readmissions for medical complications and unplanned reoperations for surgical complications not only increase the mortality but also place an increased socioeconomic burden on healthcare systems and families [27–32, 34, 35]. Previous studies have found that advanced age not only is related to increased mortality but also is associated with increased complication rates after hip fracture [1, 2, 4–8].

The rates of mortality at 1 year after hip fracture in nonagenarians have been shown to range from 24 to 51% [9–26]. The complication rates after hip fracture in nonagenarians have not been well investigated [11, 15]. Among the studies that have been conducted, the 2-years complication rate ranged from 30–50% [11, 15]. However, the sample sizes of those studies on hip fracture among nonagenarians were relatively small (*n* = 32–919) and thus could not precisely quantify all the complication rates after hip fracture among nonagenarians [9–26]. Osteoporotic hip fracture is associated with increased risk of mortality. However, death is a competing risk for complications, which makes it inappropriate to estimate complication rates in the study population with a large proportion of death during the follow-up time using traditional survival analytical methods [36–38]. Most previous studies on long-term complication rates after surgery for hip fracture among nonagenarians did not consider death of these patients as a competing risk, resulting in overestimation of the complication rates [9–26]. Therefore, we applied a competing risk analysis using data from a nationwide population database in Taiwan to estimate the mortality, readmission and reoperation rates after surgery for hip fracture among nonagenarians.

## Methods

### Data source and subjects

Data on patients in this study were retrospectively collected from two sources, namely the National Health Insurance (NHI) Database and the National Register of Deaths Database maintained by Taiwan’s Ministry of Health and Welfare. The NHI Database consists of standard computerized claims documents submitted by medical institutions seeking reimbursement through the National Health Insurance (NHI) program from 1996 to the present. The NHI program started in 1995 and as of 2012 covers the medical needs for more than 23 million people, representing more than 99% of the population of Taiwan. The completeness and accuracy of the NHI database exceed 98–99% and are guaranteed by Taiwan’s Ministry of Health and Welfare and the Bureau of National Health Insurance. All subjects in this study were 90 or more years old (nonagenarians) who received surgery for hip fracture during the period 1 January 1997 – 31 December 2010. As described previously [39], subjects were identified from the database with (a) first discharge disease codes of hip fracture [based on International Classification of Disease, Ninth Revision, Clinical Modification (ICD9-CM) codes 820, 820.0, 820.00, 820.01, 820.02, 820.09, 820.8, 820.03, 820.2, 820.20, and 820.21] and (b) procedure codes with surgery of internal fixation or hemiarthroplasty (based on ICD9-CM codes 79.15, 79.35, and 81.52). The first admission date for hip fracture was defined as the index day of surgery. The exclusion criteria included subjects with pathological fractures (ICD9-CM codes 733.14 and 733.15) or open hip fractures (ICD9-CM codes 820.1, 820.10, 820.11, 820.12, 820.19, and 820.9). Subjects who underwent surgery for injuries to the pelvis, femur, or hip region before the index day were also excluded to avoid confounding effects. Comorbidities of each subject were evaluated before or at the time of the index day according to the Charlson comorbidity index (CCI) [40]. All subjects were followed until the end of 2012, exiting the NHI program, or death.

### Outcomes of interest

We estimated three possible outcomes: (a) overall cumulative mortality; (b) cumulative incidence of the first reoperation due to surgical complications, and (c) cumulative incidence of the first medical readmission within 90 days due to medical complications after the index surgery. The duration from the index day to the day of death was defined as the overall survival time. Subjects who were alive at the end of the study or who were lost to follow-up were censored. The duration from the index day to the day of the first postoperative unplanned reoperation for surgical complications was defined as the first reoperation time. The indications for reoperation included conversion to/revision arthroplasty, surgical site infection, removal of implant due to complications, mechanical complication (including loss reduction, screw back out or cutting out, skin irritation, and implant broken/failure), dislocation, avascular necrosis of the femoral head, second hip fracture, and malunion/nonunion during the follow-up period.

The duration from the index day to the day of the first medical complication within 90 days after the index surgery was defined as the first medical readmission. Complications occurring within 90 days after the index surgery which required extra days of hospital stay or readmission to hospital for treatments included stroke, acute myocardial infarction, pulmonary embolism, acute renal failure, or acute respiratory failure. There is no clear cut-off point between medical complications and newly developed comorbidities after major surgery. We, therefore, interpreted a shorter duration from the index day to the day of the first medical readmission as indicating a greater probability that the readmissions were caused directly or indirectly by the surgery.

### Statistical analysis

The overall survival rates were estimated using the Kaplan-Meier (K-M) analysis. The log-rank test and multivariate Cox’s proportional hazards model were applied to estimate the effects of risk factors on survival time. The mortality rate in our cohort was very high; therefore, standard survival analysis was not appropriate for examining the medical and surgical complication rates. An important independent censoring assumption of standard survival analysis implies that patients who are censored at a certain time point should be representative for those still at risk at that point in time. However, because subjects who died could not have any outcomes of interest, the death could not be referred to as “censored” for the standard survival analysis. Death is referred to as a “competing risk” for the events of interest other than mortality. When the proportion of deaths is large and increases with follow-up time, the cumulative medical complication and reoperation rates will be overestimated based on the traditional K-M procedure. It is important to sufficiently account for the competing risk of death in the analysis. Therefore, we estimated the cumulative incidence of first readmission or reoperation based on the cumulative incidence function of competing risk analysis, in which death was considered as a competing risk to analyze the readmission or reoperation time [36–38]. The Gray’s test and the Fine and Gray’s model, i.e., a multivariate subdistribution hazards model based on competing risk analysis, were applied to evaluate the effects of risk on readmission and reoperation. The risk factors evaluated in those models included age, sex, fracture type, operation type, and CCI. All data management and analysis were performed using the statistical package SAS 9.4 (SAS Institute, Cary, NC) and R 3.2.2 [available at http://www.R-project.org/, R Development Core Team (2015), R: A language and environment for statistical computing. R Foundation for Statistical Computing, Vienna, Austria, especially the R libraries survival, cmprsk, and mstate].

## Results

**Fig. 1:**
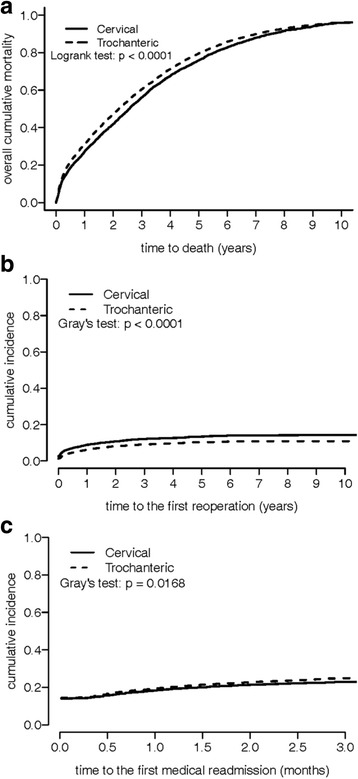
Ten-year cumulative incidence curve of (**a**) mortality stratified by fracture type, (**b**) first reoperation stratified by fracture type, (**c**) 3-months cumulative incidence curves of first medical readmission stratified by fracture type

A total of 11,184 nonagenarians who underwent surgery as primary treatment for hip fracture during the period 1997–2010 satisfied the inclusion criteria and were enrolled in the study. The cohort comprised 7,560 (67.6%) women and 3,624 (32.4%) men. Among those patients, 4,411 (39.4%) had a cervical fracture and 6,773 (60.6%) had a trochanteric fracture. Primary treatment included internal fixation in 7,264 (64.9%) and hemiarthroplasty in 3,920 (35.1%) patients (Table 1). Trochanteric fracture was slightly more prevalent than cervical fracture in women. The median survival times were 2.35 years (95% CI: 2.28–2.42 years) for nonagenarians, 2.56 years (95% CI: 2.47–2.65 years) for patients with cervical fracture, and 2.198 years (95% CI: 2.12–2.288 years) for patients with trochanteric fracture. The mortality rates were 5.5% at 1 month, 13.8% at 3 months, 20.3% at 6 months, 29.5% at 1 year, 45.0% at 2 years, 78.1% at 5 years and 95.9% at 10 years. The 1-, 2-, 5-, and 10-years cumulative incidence rates of the first reoperation were 7.3, 9.2, 11.6, and 12.2%, respectively, while those for the first medical readmission at 1 and 3 months were 18.9 and 24.1%, respectively (Table 2 and Fig. 1). Cumulative mortality and cumulative readmission rates were significantly higher and the cumulative reoperation rate was significantly lower in patients with trochanteric fracture than in those with cervical fracture (Table 2 and Fig. 1).

**Table 1:** Baseline characteristics of hip fractures among nonagenarians stratified by fracture type

		Total		Cervical		Trochanteric		
		*N* = 11,184		*N* = 4,411	(39.4%)	*N* = 6773	(60.6%)	
		*N*	(%)	*N*	(%)	*N*	(%)	*p*-value
Age (yrs)	Mean ± SD	93	(2.7)	93	(2.4)	93	(2.9)	<0.001
Gender	Female	7,560	(67.6)	3,022	(68.5)	4,538	(67.0)	0.098
	Male	3,624	(32.4)	1,389	(31.5)	2,235	(33.0)	
Operation Type	Fixation	7,264	(64.9)	758	(17.2)	6,506	(96.1)	<0.001
	Hemiarthroplasty	3,920	(35.1)	3,653	(82.8)	267	(3.9)	
CCI^a^ Score	0	4,671	(41.8)	1,865	(42.3)	2,806	(41.4)	0.049
	1	2,707	(24.2)	1,089	(24.7)	1,618	(23.9)	
	2	1,580	(14.1)	571	(12.9)	1,009	(14.9)	
	3	983	(8.8)	403	(9.1)	580	(8.6)	
	≧4	1,243	(11.1)	483	(10.9)	760	(11.2)	
Hypertension	No	6,017	(53.8)	2,318	(52.6)	3,699	(54.6)	0.033
	Yes	5,167	(46.2)	2,093	(47.4)	3,074	(45.4)	
Diabetes Mellitus	No	9,847	(88.0)	3,911	(88.7)	5,936	(87.6)	0.107
	Yes	1,337	(12.0)	500	(11.3)	837	(12.4)	
Heart Disease	No	8,296	(74.2)	3,300	(74.8)	4,996	(73.8)	0.216
	Yes	2,888	(25.8)	1,111	(25.2)	1,777	(26.2)	
Chronic Pulmonary Disease	No	8,570	(76.6)	3,429	(77.7)	5,141	(75.9)	0.025
	Yes	2,614	(23.4)	982	(22.3)	1,632	(24.1)	
Cerebrovascular Disease	No	9,377	(83.8)	3,708	(84.1)	5,669	(83.7)	0.618
	Yes	1,807	(16.2)	703	(15.9)	1,104	(16.3)	
Cancer	No	10,353	(92.6)	4,069	(92.2)	6,284	(92.8)	0.302
	Yes	831	(7.4)	342	(7.8)	489	(7.2)	
Primary solid cancer	No	10,397	(93.0)	4,082	(92.5)	6,315	(93.2)	0.162
	Yes	787	(7.0)	329	(7.5)	458	(6.8)	
Chronic Liver Disease	No	10,756	(96.2)	4,231	(95.9)	6,525	(96.3)	0.267
	Yes	428	(3.8)	180	(4.1)	248	(3.7)	
Chronic Renal Disease	No	10,782	(96.4)	4,232	(95.9)	6,550	(96.7)	0.038
	Yes	402	(3.6)	179	(4.1)	223	(3.3)	

*SD* standard deviation

^a^Charlson Comorbidity Index

**Table 2:** Cumulative mortality rates and cumulative incidence of first reoperation or readmission after surgery for hip fracture among nonagenarians stratified by fracture type

				Cumulative Incidence					
	Mortality			Reoperation (Surgical Complication)			Readmission (Medical Complication)		
Time	Total	Cervical	Trochanteric	Total	Cervical	Trochanteric	Total	Cervical	Trochanteric
1-month	5.50%	4.60%	6.10%	2.80%	4.10%	2.00%	18.90%	18.30%	19.20%
3-month	13.80%	12.50%	14.60%	4.50%	5.90%	3.50%	24.10%	22.90%	24.90%
6-month	20.30%	18.50%	21.60%	5.60%	7.10%	4.60%			
1-year	29.50%	27.10%	31.10%	7.30%	9.00%	6.20%			
2-year	45.00%	41.80%	47.10%	9.20%	10.80%	8.10%			
5-year	78.10%	75.80%	79.60%	11.60%	13.50%	10.30%			
10-year	95.90%	95.90%	95.90%	12.20%	14.30%	10.90%

We evaluated the effects of risk factors on survival using multivariate survival analysis. Age, male gender, trochanteric fracture, and higher CCI were significant risk factors for mortality. For each 1-year increase in age, the hazard ratio (HR) increased by 1.015 (95% CI: 1.012–1.020). Men had a 31% (HR, 1.31; 95% CI: 1.26–1.37) higher risk of overall death than women. Trochanteric fracture had a 1.14 times (95% CI: 1.06–1.23) higher hazard ratio (HR) of overall death than cervical fracture. Patients with a CCI of one or more were at markedly greater risk for overall death than patients without comorbidities (CCI = 1, HR 1.12, 95% CI: 1.06–1.18; CCI = 2, HR 1.20, 95% CI: 1.13–1.28; CCI = 3, HR 1.30, 95% CI: 1.20–1.40; CCI ≥ 4, HR 1.55, 95% CI: 1.45–1.66, Table 3).

**Table 3:** (a) Hazard ratios of the risk factors associated with death from cause-specific hazard model based on multivariate Cox’s model, (b) subdistribution hazard ratio of the risk factors associated with the first reoperation, and (c) readmission based on multivariate Fine and Gray’s (subdistribution hazard ratio, sub-HR) model from competing risk analysis

		(a) Death			(b) Reoperation			(c) Readmission		
		HR	(95%C.I.)	*p*-value	sub-HR	(95%C.I.)	*p*-value	sub-HR	(95%C.I.)	*p*-value
Age	Year	1.015	(1.012–1.019)	<0.001	0.98	(0.95–0.99)	0.041	1.02	(1.01–1.03)	0.003
Gender	Female	(REF)			(REF)			(REF)		
	Male	1.31	(1.26–1.37)	<0.001	0.92	(0.82–1.04)	0.182	1.71	(1.59–1.84)	<0.001
Fracture	Cervical	(REF)			(REF)			(REF)		
	Trochanteric	1.14	(1.06–1.23)	<0.001	0.72	(0.59–0.89)	0.002	1.08	(0.95–1.22)	0.257
Operation	Fixation	(REF)			(REF)			(REF)		
	Hemiarthroplasty	1.03	(0.96–1.11)	0.371	0.96	(0.78–1.18)	0.665	1.00	(0.88–1.14)	0.998
CCI^a^	0	(REF)			(REF)			(REF)		
	1	1.12	(1.06–1.18)	<0.001	0.94	(0.82–1.08)	0.383	2.63	(2.37–2.91)	<0.001
	2	1.20	(1.13–1.28)	<0.001	0.97	(0.82–1.14)	0.698	3.02	(2.71–3.37)	<0.001
	3	1.30	(1.20–1.40)	<0.001	1.06	(0.87–1.29)	0.570	3.39	(2.99–3.85)	<0.001
	4	1.55	(1.45–1.66)	<0.001	0.80	(0.65–0.97)	0.025	3.60	(3.15–4.13)	<0.001

^a^Charlson Comorbidity Index

We evaluated the effects of risk factors on the time to the first reoperation with a multivariate subdistribution proportional hazards model. The results revealed that older age, cervical fracture, and higher CCI were significant risk factors for reoperation. After controlling for age, gender, and comorbidities, trochanteric fracture had a 0.72 times (95% CI: 0.59–0.89) lower sub-HR for reoperation than fixation. We also explored the effects of risk factors on the time to the first readmission due to medical complications and found that age, male gender, and higher CCI score were significant risk factors for readmission within 90 days after surgery (Table 3). Trochanteric fracture had a similar risk for readmission than cervical fracture.

Medical and surgical complications during follow-up are common after treatment for hip fracture, especially in patients of advanced age. In this study, 2,694 (24.1%) patients had at least one medical complication within 3 months after the index surgery, with the most common complications being acute pulmonary disorders (such as acute exacerbation of chronic obstructive pulmonary disease, 48.37%; pneumonia, 43.50%; acute respiratory failure, 30.59%) and acute renal failure (11.47%) (Table 4). Of the 2,694 patients who were readmitted for medical complications within 3 months after the index surgery, 36.5% died within 3 months of readmission (data not shown in the tables).

**Table 4:** Causes of the first reoperations or readmission after surgery for hip fracture stratified by fracture type

(1) Reoperation																					
	1-Month			3-Month			6-Month			1-Year			2-Year			5-Year			10-Year		
Fracture type^a^	Total	Cervical	Troch	Total	Cervical	Troch	Total	Cervical	Troch	Total	Cervical	Troch	Total	Cervical	Troch	Total	Cervical	Troch	Total	Cervical	Troch
N^b^	328	184	144	509	263	246	634	317	317	818	397	421	1027	477	550	1253	576	677	1295	596	699
Conversion to/revision arthroplasty^c^	17.38%	21.74%	11.81%	26.13%	26.24%	26.02%	32.49%	33.12%	31.86%	35.45%	37.03%	33.97%	37.39%	40.46%	34.73%	36.63%	41.15%	32.79%	36.37%	41.11%	32.33%
Infection	37.20%	45.65%	26.39%	31.24%	38.40%	23.58%	28.55%	33.75%	23.34%	24.69%	29.22%	20.43%	21.52%	26.00%	17.64%	19.07%	22.05%	16.54%	18.61%	21.31%	16.31%
Removal of implant	11.89%	7.61%	17.36%	22.79%	12.93%	33.33%	25.87%	12.93%	38.80%	25.92%	14.86%	36.34%	23.66%	13.84%	32.18%	21.07%	12.33%	28.51%	20.62%	12.08%	27.90%
Mechanical complication^d^	23.17%	23.37%	22.92%	33.20%	29.28%	37.40%	33.12%	26.50%	39.75%	30.81%	25.19%	36.10%	26.97%	22.64%	30.73%	23.46%	19.44%	26.88%	23.09%	19.46%	26.18%
Dislocation	13.11%	20.65%	3.47%	12.38%	21.29%	2.85%	10.25%	17.98%	2.52%	8.31%	14.86%	2.14%	6.62%	12.37%	1.64%	5.43%	10.24%	1.33%	5.25%	9.90%	1.29%
Avascular necrosis	3.96%	1.63%	6.94%	2.55%	1.14%	4.07%	2.05%	0.95%	3.15%	1.59%	0.76%	2.38%	1.27%	0.63%	1.82%	1.04%	0.52%	1.48%	1.00%	0.50%	1.43%
Second hip fracture	30.18%	22.83%	39.58%	33.01%	29.28%	36.99%	37.54%	37.22%	37.85%	45.35%	44.58%	46.08%	53.16%	51.99%	54.18%	59.46%	59.20%	59.68%	60.46%	60.23%	60.66%
Malunion	0.00%	0.00%	0.00%	0.00%	0.00%	0.00%	0.00%	0.00%	0.00%	0.00%	0.00%	0.00%	0.10%	0.00%	0.18%	0.16%	0.00%	0.30%	0.15%	0.00%	0.29%
Nonunion	0.61%	0.00%	1.39%	2.16%	1.14%	3.25%	3.31%	2.21%	4.42%	4.77%	3.53%	5.94%	4.28%	2.94%	5.45%	3.91%	2.60%	5.02%	3.78%	2.52%	4.86%
(2) Medical Readmission																					
	1-Month			3-Month																	
Fracture type^a^	Total	Cervical	Troch	Total	Cervical	Troch															
N^a^	2109	806	1303	2694	1008	1686															
Stroke	6.83%	7.32%	6.52%	7.94%	8.33%	7.71%															
Acute myocardial infarction	3.18%	3.47%	2.99%	3.19%	3.67%	2.91%															
Pulmonary embolism	1.38%	1.12%	1.53%	1.19%	0.89%	1.36%															
Deep vein thrombosis	1.28%	0.74%	1.61%	1.37%	0.99%	1.60%															
Acute renal failure	10.05%	10.42%	9.82%	11.47%	12.60%	10.79%															
Acute respiratory failure	25.27%	22.33%	27.09%	30.59%	26.39%	33.10%															
Pneumonia	37.08%	36.72%	37.30%	43.50%	41.27%	44.84%															
Exacerbation of COPD	52.82%	52.61%	52.95%	48.37%	48.21%	48.46%															

^a^ Fracture type : *Cervical* Cervical fracture, *Troch* Trochanteric fracture

^b^ *N* = The number of subjects who had at least one readmission or reoperation

^c^ % = Percentage of subjects who had a certain cause of complication among the total number of subjects who had at least one complication. Subjects might have had more than one readmission or operation due to multiple causes

^d^ Mechanical complication included loss reduction, screw back out or cutting out, skin irritation, and implant broken/failure

A total of 1,027 nonagenarians had at least one reoperation within 2 years after the index surgery. Among those who had reoperations within 2 years after the index surgery, most reoperations were caused by second hip fracture (53.16%), conversion to/revision anthroplasty (37.39%), mechanical complication (26.97%, including loss reduction, screw back out or cutting out, skin irritation, and implant broken/failure), removal of implant (23.66%), and infection (21.52%) (Table 4). Of the patients who required a second surgery, 33.7% died within 1 year after reoperation (data not shown in the tables).

## Discussion

To the best of our knowledge, this is the first population study to use competing risk analysis to explore mortality and readmission due to medical complications as well as reoperation due to surgical complications after hip fracture among nonagenarians based on data from a nationwide database. A number of meta-analyses have revealed that age is an important risk factor for death in elderly adults after hip fracture [4, 6, 7]. In this study, we investigated the outcomes of 11,184 nonagenarians after hip fracture surgery in Taiwan. We found that the mortality rates were 5.5% at 1 month, 13.8% at 3 months, 20.3% at 6 months, 29.5% at 1 year, 45.0% at 2 years, 78.1% at 5 years, and 95.9% at 10 years after surgery. Previous studies reported that the 1-, 3-, 6-months, and 1-year mortality rates ranged from 5.9 to 26.4%, 15 to 36%, 24.7 to 42%, and 24 to 51%, respectively [9–26]. The short-term mortality rates in Taiwan were lower than those in New Zealand, Spain, Scotland, Netherlands, Canada, and the United States; but higher than those in Japan and Korea [9–26]. Previous studies reported that the short-term mortality rates among nonagenarians varied from region to region [9–26]. Japan, Korea, and Taiwan seem to have lower short-term mortality rates after hip fracture among nonagenarians than those of Western countries. However, the sample sizes in the previous studies were relatively small (from 32 to 919 nonagenarians) compared to that in our study [9–26]. Differences in findings among the studies from different countries might be due to differences in selection criteria, distributions of gender-stratified nonagenarians in the populations, distributions of smoking status, physical activity, bone mineral density, nutrition, hip strength, and comorbidities, in addition to sample sizes.

In a review on hip fracture-related mortality among elderly adults from 1996 to 1998, Haleem et al. found that the rates of mortality ranged from 11 to 23% at 6 months and from 22 to 29% at 1 year [3]. In a meta-analysis of mortality after hip fracture in patients of advanced age, Abrahamsen et al. found that the crude mortality rates ranged from 3.3 to 17.2% at 1 month, 6.4 to 20.4% at 3 months, 7.1 to 23% at 6 months, and 5.9 to 59% at 1 year [4]. They also found that excess mortality was highest in the first 6 months and that it decreased after 1 year, but remained high throughout the next several years. Several studies have shown that hip fractures affected short-term but not long-term mortality among elderly adults [1, 8, 12, 13, 24, 41]. However, others have shown that there is a gradual increase in mortality for up to 5 years after fracture [1, 41–46]. The 1-, 2-, 5-years, and 10-years mortality rates were 29.5, 45.0, 78.1, and 95.9% in this study. We found that the first-year mortality of nonagenarians after hip fracture was similar to that of elderly adults [1–8]. However, after the first year, the mortality rates increased more rapidly among nonagenarians after hip fracture than those among other elderly age groups in other studies [15, 26]. Nonagenarians, due to their advanced age, naturally have a very high mortality rate, irrespective of any past history of hip fracture. Whether excess mortality caused by hip fracture among nonagenarians increases persistently for several years after hip fracture remains unclear. We believe that hip fracture and aging both continue to have effects on mortality 1 year after fracture among nonagenarians.

The results of our multivariate survival analysis showed that age, male gender, and higher CCI were significant predictors of mortality after hip fracture in nonagenarians. In a meta-analysis, Hu et al. reported that the yearly HR for age was 1.05 among elderly adults after hip fracture [7]. In our study, the estimated yearly HR for age was 1.015 (95% CI: 1.012–1.020). The age effect on mortality in nonagenarians was not as high as in elderly adults. Several previous studies also did not find that age was a risk factor for mortality among nonagenarians [12, 17]. One possible reason for the discrepancy is that our patient population (>90 years) was more homogeneous than the populations included in other population-based studies (>65 years) on predictors of mortality after fracture.

We found that men were at 31% greater risk for mortality than women (HR, 1.31, 95% CI: 1.26–1.37). Many meta-analyses have found that elderly men are at higher risk for mortality than elderly women after hip fracture [4, 6, 7]. Hu et al. estimated that men had a 70% greater risk of overall death than females (HR 1.70; 95% CI: 1.51–1.04) [7]. Although several studies have reported that male nonagenarians have higher mortality rates than their female counterparts [16, 17, 22, 26], other studies have shown that male gender is not a significant predictor of mortality among nonagenarians [12, 20, 25]. In our study, the HR of male gender in nonagenarians was not as high as that of other age subgroups of elderly adults. Males had more comorbidities, specifically chronic obstructive pulmonary disease, than females in our study. The higher prevalence of multiple comorbidities in males may explain the higher risk. However, in a number of population-based studies, men had higher mortality rates and died earlier than women; therefore, nonagenarians represent a more homogeneous population in which more healthy males survived compared with the younger elderly adults. This explains, at least in part, why the effect of male gender in nonagenarians was not as high as that in the younger elderly subgroups, and may further suggest why some studies did not find that male gender was a predictor of mortality after hip fracture among nonagenarians. The small sample sizes and shorter follow-up durations might also have contributed to some of the differences in the previous studies.

We found that trochanteric fracture was associated with a 14% greater likelihood of death than cervical fracture (HR, 1.14: 95% CI: 1.06–1.23). Previous studies on elderly adults after hip fracture have also reported that trochanteric fracture is associated with higher risk for mortality than cervical fracture [2, 47–52]. However, the association between fracture type and mortality in nonagenarians is not consistent among studies [11, 12, 14, 17, 20, 22, 23, 25]. Most studies on hip fracture in nonagenarians have reported that mortality rates are similar between trochanteric fracture and cervical fracture [11, 12, 17, 20, 23, 25]. Only Ishida et al. and Kang et al. demonstrated that trochanteric fracture was associated with a higher risk for mortality than cervical fracture among nonagenarians [14, 22]. In this study, patients with trochanteric fracture were older and nonagenarians had more comorbidities than those reported in studies on younger elderly adults. Gender and age may have a mixed effect on risk of trochanteric fracture.

We found no significant difference between internal fixation and arthroplasty in mortality among nonagenarians, a finding consistent with that reported by Takamine et al. and Tay et al. [20, 25]. However, Ishida et al. demonstrated that hemiarthroplasty was a significant risk factor for mortality among 74 nonagenarians [14]. In our study, most (82.8%) patients with cervical fracture received hemiarthroplasty and most (96.1%) with trochanteric fracture received internal fixation. Natural causes of death among nonagenarians and unbalanced mixed subject-variable distributions between types of operations and fractures all contributed to the reasons for the non-significant differences between internal fixation and arthroplasty for mortality.

In this study, higher CCI was associated with higher risk of mortality among nonagenarians. Several studies have explored the relationship between comorbidities and mortality after hip fracture among nonagenarians and many of the results varied from study to study [15, 16, 19, 20, 26]. Two studies found that a larger number of comorbidities increased mortality [16, 19] and three studies reported no association between comorbidities and mortality after hip fracture among nonagenarians [15, 20, 26]. Many previous studies have explored whether American Society of Anesthesiologist (ASA) score is predictive of mortality after hip fracture among nonagenarians, but the results were not conclusive [12, 14–18, 22, 23, 25]. Some studies found that a higher ASA score was associated with mortality [12, 14, 16, 18]. However, other studies did not find that ASA score was associated with mortality after hip fracture among nonagenarians [15, 17, 22, 23, 25]. Aging, higher CCI and higher ASA scores are all associated with a larger number of and more severe comorbidities. Differences in competing risk of death, confounding variables, such as age, CCI, ASA score, and sample size, may interact with one another within studies resulting in different conclusions among studies about the effects of comorbidities. However, there is still no agreement among researchers as to how comorbidities should be measured and how the measurement should be evaluated statistically.

Postoperative readmission and reoperation rates increase the use of healthcare resources and are often indicative of healthcare quality for hip fractures. Major medical complications result in readmission, whereas major surgical complications result in reoperation. Several studies on outcomes after surgery for hip fracture in patients ≥ 65 years revealed that the readmission rate within 3 months varied from 10 to 20%, the 1-year reoperation rate varied from 7 to 13% and the 2-years reoperation rate varied from 15 to 28% [27–32, 34, 35]. To the best of our knowledge, only two studies have reported on the readmission and reoperation rates after hip fracture among nonagenarians [11, 15]. Those studies found that the 2-years complication rates were as high as 30–50% [11, 15]. Osteoporotic hip fracture, aging, and comorbidities all contributed to excess mortality. If subjects died, these subjects could not have any complications after their death. Readmission or reoperation rate cannot be estimated without considering competing death, which may cause overestimation of the risk, especially for nonagenarians. Using traditional Kaplan-Meier method, the cumulative medical complication and reoperation rates would be overestimated. Kaplan-Meier estimates are biased because the death rate is large and will increase with the follow-up time. Therefore, to obtain a more valid estimate of the risk for medical or surgical complication, we applied a competing risk analysis to estimate the cumulative incidence of the readmission and reoperation [36, 37]. We found that the 1-, 2-, and 5-years cumulative incidence rates of the first reoperation were 7.3, 9.2, and 11.6% for the nonagenarians, respectively. Moreover, the 1- and 3-months cumulative incidence rates of the first medical readmission were 18.9 and 24.1%, respectively, for the nonagenarians in Taiwan. Because competing death was not considered, previous studies had higher long-term reoperation rates than those of the current study [36, 37]. Our finding implies that nonagenarians appeared to have a higher all-cause hazard ratio for death, had lower risks for various surgical complications when we considered the competing death in our analysis. Therefore, nonagenarians had a slightly lower reoperation rate than those in the previous studies. However, even under this circumstance, male nonagenarians still had slightly higher short-term readmission rates of medical complications. The competing risk of death had less effect on the 3-months readmission rates than on the long-term reoperation rates. Nonagenarians did not die quickly due to aging during the 3-months follow-up after hip fracture. Therefore, nonagenarians in our study had similar short-term readmission rates after hip fracture as nonagenarians in the previous studies.

In this study, predictors of mortality included older age, male gender, trochanteric fracture, and higher CCI score. However, trochanteric fracture correlated with a low risk for reoperation (Table 3). After controlling for age, gender, operation type, and comorbidities, we found that patients with trochanteric fracture were at 14% greater risk for death than those with cervical fracture (HR = 1.14, 95% CI: 1.06–1.23) but that their risk of reoperation was 28% lower than patients with cervical fracture (HR = 0.72; 95% CI: 0.59–0.89). This finding implies that nonagenarians with trochanteric fracture appeared to have a higher all-cause hazard for death but lower risks of reoperation than those with cervical fracture. When the follow-up time was short, the cumulative mortality rate was still low; many subjects with trochanteric fracture still had a higher hazard ratio for 90-days readmission (sub-HR = 1.08) than those with cervical fracture. As described in the previous study [39], during the first 3-months follow-up, the short-term death rate was small, subjects with trochanteric fracture had larger readmission hazard rate (sub-HR = 1.08) than subjects with cervical fracture. In contrast, after 6-months follow-up, there were fewer subjects with trochanteric fracture during follow-up but they were healthier and therefore exposed to less risk for long-term surgical complications. Thus, subjects with trochanteric fracture had a smaller risk (sub-HR = 0.72) for surgical reoperation than those with cervical fracture. When a population had much higher mortality rate than complication rate during the early phase of follow-up, using high cost but more benefit treatments to reduce the long-term surgical complication would be limited. When the competing hazards of a risk factor on mortality and complication are different, it raises the difficulty of conducting analysis and interpretation. A cost-effective analysis from a competing risk viewpoint may be quite different from that without considering the competing death. In practice, competing risk analysis is a useful tool to address the actual risk and to provide a more realistic result to make a decision.

The competing death phenomenon not only affected the effect of fracture type, but also slightly affected other factors, such as age, gender, and operation type, during long-term follow-up after surgery in our study. Moreover, the competing death phenomenon may have no effect on short-term follow-up after surgery. As older age, male gender, trochanteric fracture, and higher CCI were significant risk factors for mortality, age, male gender, and higher CCI also had higher risks for medical readmission within the 3-months follow-up period after surgery (Table 3).

Most readmissions occur within 3 months [30, 35]. Some studies also found that readmissions or reoperations were associated with higher mortality within 1 year [27, 28, 31, 32, 34, 35]. We found that 984 (36.5%) of 2,694 nonagenarians died within 3 months after the first medical complications and that 859 (33.7%) of 1,296 nonagenarians died within 1 year after the first reoperation. Jennings and de Boer found that 12 (37.5%) of 32 nonagenarians who had surgical complications died within 6 months, and 17 (38.6%) of 44 who had medical complications died within 6 months [11]. Our analysis of nonagenarians revealed similar findings to those of previous studies on elderly adults after hip fractures [11, 28, 31–34]. Prevention of hip fracture among nonagenarians is likely the best way to reduce the high risk effect of aging on mortality, readmission, and reoperation after surgery.

The overall 3-months, 1- and 5-years mortality rates among nonagenarians in Taiwan were around 14, 30 and 78%, respectively. The 1- and 5-years reoperation rates were around 7 and 12%, respectively. The 30- and 90-days medical complication rates were around 19 and 24%, respectively. Once nonagenarians had the first readmission, 36.5% died within 3 months after medical complications. Once nonagenarians had the first reoperation, 33.7% died within 1 year after reoperation (data not shown in the tables). The impact of medical complications on mortality among the nonagenarians was extraordinarily high. Therefore, postoperative care to prevent medical complications should be the most effective strategy to reduce the mortality rates among nonagenarians with hip fracture. However, prevention of hip fracture in this patient population is the best way to reduce mortality, readmission and reoperation after surgery.

### Limitations

All of the patients in this study were nonagenarians who underwent surgical treatment for hip fracture; however, there was great variation in follow-up period among the patients (2–11 years). The NHRI database differs from the hip fracture registry database in that the former does not record all clinical information [2, 53]. Therefore, some unknown clinical confounding factors might have existed or changed during the follow-up period. In addition, we did not adjusted for many of the risk variables, such as general condition prior to surgery, smoking and alcohol behavior, BMI, bone mineral density, comorbidities, and quality of life. Agreement about how comorbidities should be evaluated and how the measures of comorbidities should be placed into a statistical model is still absent. Aging, higher ASA cores, delayed surgery, and higher CCI are associated with one another. For example, a poor ASA score is caused by a large number of comorbidities, which implies that more time is needed to stabilize the medical comorbidities, and further suggests a long waiting time for surgery, as well as poor outcomes after surgery. We used the CCI to represent the combined severity of multiple comorbidities, which has been shown to be a key consideration in hip fracture [54, 55]. In addition, we used ICD9-CM codes to define the medical complications and reasons for reoperation, which might have lacked the precision needed for a precise evaluation of the estimated readmission and reoperation rates. Finally, the readmission rates and reoperation rates might also vary depending on the definitions used. Therefore, caution should be taken in extrapolating our results.

## Conclusions

During the period 1997–2010, 11,184 nonagenarians received surgery for hip fracture in Taiwan. The 3-months postoperative mortality and medical readmission rates were 13.8 and 23.8%, respectively, and the 2-years mortality and reoperation rates were 45.0 and 9.2%, respectively. Among nonagenarians who were readmitted for the first time, 36.5% died within 3 months. Among nonagenarians who received their first reoperation, 33.7% died within 1 year. High complication rates caused excess mortality. Postoperative care to prevent medical complications is an important strategy to reduce the mortality rates in nonagenarians with hip fracture.

## Abbreviations

ASA: American Society of Anesthesiologists
CCI: Charlson comorbidity index
HR: Hazard ratio
ICD9-CM: International classification of disease, ninth revision, clinical modification
KM: Kaplan-Meier
NHI: National health insurance
sub-HR: subdistribution hazard ratio
